# Identification and characterization of a novel β-lactamase gene, *bla*_AMZ–1_, from *Achromobacter mucicolens*

**DOI:** 10.3389/fmicb.2023.1252427

**Published:** 2023-09-21

**Authors:** Yuan Zhang, Jingxuan Zhao, Guozhi Zhang, Naru Lin, Yuning Sha, Junwan Lu, Tingting Zhu, Xueya Zhang, Qiaoling Li, Hailin Zhang, Xi Lin, Kewei Li, Qiyu Bao, Dong Li

**Affiliations:** ^1^The Second Affiliated Hospital and Yuying Children’s Hospital, Wenzhou Medical University, Wenzhou, China; ^2^Key Laboratory of Medical Genetics of Zhejiang Province, Key Laboratory of Laboratory Medicine, Ministry of Education, School of Laboratory Medicine and Life Sciences, Wenzhou Medical University, Wenzhou, China; ^3^Medical Molecular Biology Laboratory, School of Medicine, Jinhua Polytechnic, Jinhua, China

**Keywords:** *bla*_AMZ–1_, AmpC, *Achromobacter mucicolens*, resistance gene, kinetic analysis

## Abstract

**Background:**

*Achromobacter* is a genus of gram-negative bacteria that can act as opportunistic pathogens. Recent studies have revealed that some species of *Achromobacter* show inherent resistance to β-lactams, but the resistance mechanisms of *Achromobacter mucicolens* have rarely been reported.

**Method:**

The bacterium was isolated using standard laboratory procedures. The agar dilution method was used to determine the minimum inhibitory concentrations (MICs). Genome sequencing was performed using the PacBio RS II and Illumina HiSeq 2500 platforms, and the Comprehensive Antibiotic Resistance Database (CARD) was used to annotate the drug resistance genes. The localization of the novel β-lactamase AMZ-1 was determined, and its characteristics were determined via molecular cloning and enzyme kinetic analysis. The phylogenetic relationship and comparative genomic analysis of the resistance gene-related sequences were also analyzed.

**Result:**

*Achromobacter mucicolens* Y3, isolated from a goose on a farm in Wenzhou, showed resistance to multiple antibiotics, including penicillins and cephalosporins. *Bla*_AMZ–1_ showed resistance to amoxicillin, penicillin G, ampicillin, cephalothin and cefoxitin, and the resistance activity could be inhibited by β-lactamase inhibitors. Enzyme kinetic analysis results showed that AMZ-1 has hydrolytic activity against a wide range of substrates, including cephalothin, amoxicillin, penicillin G, and cefoxitin but not ampicillin. The hydrolytic activity of AMZ-1 was greatly inhibited by avibactam but much more weakly inhibited by tazobactam. Mobile genetic elements could not be found around the *bla*_AMZ–1_-like genes, which are conserved on the chromosomes of bacteria of the genus *Achromobacter*.

**Conclusion:**

In this study, a novel AmpC gene, *bla*_AMZ–1_, from the animal-origin bacterium *A. mucicolens* Y3 was identified and characterized. It conferred resistance to some penicillins and first- and second-generation cephalosporins. The identification of this novel resistance gene will be beneficial for the selection of effective antimicrobials to treat associated infections.

## Introduction

*Achromobacter*, a genus of the family *Alcaligenaceae*, are gram-negative and nonfermentative. This genus was originally discovered and established in 1923 by the Committee of the Society of American Bacteriologists (Isler et al., 2020). These organisms are widely distributed in the environment and have the potential to become opportunistic pathogens under certain conditions, such as cystic fibrosis, blood or solid organ malignancies, renal failure, or specific immune deficiencies (Swenson and Sadikot, 2015). Currently, there are 19 officially recognized species within the *Achromobacter* genus, and most members of this genus exhibit nonpathogenic characteristics, except *Achromobacter xylosoxidans*. *A. xylosoxidans* has been demonstrated to be an opportunistic pathogen causing a range of infections in immunocompromised individuals (Ahmed et al., 2009), while the pathogenic potential of *Achromobacter mucicolens* has been infrequently documented.

In recent years, clinical *Achromobacter* isolates have presented multidrug resistance, posing a challenge to effective infection management in affected patients (Isler et al., 2020). Efflux pumps and chromosome-encoded β-lactamases are expressed at a basal level, leading to reduced drug susceptibility. Despite the identification of several β-lactamases, such as TMB-1, OXA-114, OXA-285a, and OXA-285b, in *A. xylosoxidans* isolates (Doi et al., 2008; El Salabi et al., 2012; Papalia et al., 2018), the drug resistance mechanism of *A. mucicolens* has rarely been documented (Al-Asadi et al., 2022).

β-Lactams remain dominant among antibacterial drugs (Bush and Bradford, 2016), but the overuse of these antibiotics has led to increased microbial resistance. There are three main mechanisms of bacterial resistance to β-lactams: hydrolysis of drugs by β-lactamases (Li et al., 2007), inability of drug target binding (Li et al., 2007), and drug target alteration. The main resistance mechanism of gram-negative bacteria is the production of β-lactamases, which inactivate β-lactams by hydrolyzing the structural β-lactam ring (Li et al., 2007; Bush and Bradford, 2016). AmpC β-lactamases have a catalytic serine residue and, based on their structure, are assigned to class C in the Ambler classification of β-lactamases (Ambler, 1980). AmpC β-lactamases can hydrolyze penicillins and cephalosporins, including cephamycins (such as cefoxitin and cefotetan), oxyiminocephalosporins (such as ceftazidime, cefotaxime, and ceftriaxone), and monobactams (such as aztreonam) (Jacoby, 2009). AmpC β-lactamases have a molecular weight between 34 and 40 kDa, generally have isoelectric points > 8.0, and can be induced and expressed at high levels by many bacteria due to mutations (Jacoby, 2009). The increased antibiotic resistance resulting from overexpression of AmpC enzymes has become an increasingly serious problem, especially with regard to infections caused by opportunistic bacterial pathogens (Tamma et al., 2019).

In this study, we isolated an *A. mucicolens* strain from a goose, sequenced its complete genome sequence and designated it *A. mucicolens* Y3. The novel AmpC gene *bla*_AMZ–1_ identified in the chromosome of *A. mucicolens* Y3 conferred resistance to amoxicillin, penicillin G, ampicillin, cephalothin and cefoxitin. Finding novel resistance genes will help to further elucidate the complexity of drug resistance mechanisms.

## Materials and methods

### Bacterial strains and plasmids

*Achromobacter mucicolens* Y3 was isolated from the fecal swab of a goose at a livestock farm during a survey on the antimicrobial resistance of bacteria in Wenzhou, southern China. The strain species was verified using 16S ribosomal RNA (rRNA) gene homology (Konstantinidis and Tiedje, 2005) and average nucleotide identity (ANI) analyses (Richter and Rosselló-Móra, 2009). The bacterial strains and plasmids used in this work are listed in Table 1.

**TABLE 1:** Bacterial strains and plasmids used in this work.

Strain or plasmid	Relevant characteristic(s)	Reference or source
**Strain**		
Y3	The wild-type strain of *Achromobacter mucicolens* Y3	This study
DH5α	*Escherichia coli* DH5α was used as a host for cloning of the *bla*_AMZ–1_ gene	Our laboratory collection
BL21 (DE3)	*Escherichia coli* BL21 (DE3) was used as a host for the expression of AMZ-1	Our laboratory collection
ATCC 25922	*Escherichia coli* ATCC 25922 was used as a quality control strain for antimicrobial susceptibility test	Our laboratory collection
pUCP24-*bla*_AMZ–1_/DH5α	DH5α carrying the pUCP24 vector with the *bla*_AMZ–1_ gene with its upstream promoter region	This study
pColdI-*bla*_AMZ–1_/ BL21 (DE3)	BL21 (DE3) carrying the pColdI vector with the open reading frame of the *bla*_AMZ–1_ gene	This study
**Plasmid**		
pUCP24	Cloning vector for the PCR product of the *bla*_AMZ–1_ gene with its upstream promoter region, GEN*^r^*	Our laboratory collection
pColdI	Expression vector for the PCR product of the ORF of the *bla*_AMZ–1_ gene, AMP^r^	Our laboratory collection

^r^Resistance. GEN, gentamicin; AMP, ampicillin; ORF, open reading frame.

### Antimicrobial susceptibility testing

The standard broth dilution method was used to determine minimum inhibitory concentrations (MICs) with Mueller Hinton (MH) agar plates according to the Clinical and Laboratory Standards Institute (CLSI, 2021) guidelines. Thirteen antimicrobials, including ampicillin, amoxicillin, aztreonam, and so on were tested (Table 2). The bacterial solution cultured overnight was diluted ten-fold with normal saline after adjusting the bacterial concentration to the 0.5 McFarland’s standard and then used to determine the MICs of the antimicrobials. The CLSI (2021) guidelines were followed to determine the antibiotic sensitivity pattern based on the measured antibiotic MIC levels. *Escherichia coli* ATCC25922 was used as a standard quality control.

**TABLE 2:** Minimum inhibitory concentrations of 15 antibiotics for 5 strains (μg/mL).

Antibiotic	Y3	pUCP24-*bla*_AMZ–1_/DH5α	pUCP24/DH5α	ATCC 25922	DH5α
Ampicillin	16	32	4	8	8
Amoxicillin	64	128	2	8	4
Aztreonam	64	0.12	0.06	0.12	0.06
Cefazolin	128	1	0.5	2	1
Cefotaxime	> 256	0.06	0.06	0.06	0.06
Cefoxitin	32	8	2	2	4
Ceftazidime	1	< 0.015	0.25	0.25	1
Ceftriaxone	32	0.06	0.03	0.06	0.03
Cephalothin	> 1,024	16	< 4	8	<4
Imipenem	2	0.25	0.25	0.25	0.125
Meropenem	1	0.008	0.008	0.03	< 0.004
Penicillin G	1,024	512	8	32	16
Piperacillin	< 0.25	8	8	1	8
Piperacillin + tazobactam	< 0.25	2	1	1	2
Aztreonam + avibactam	0.03	< 0.015	<0.015	0.06	0.03

### Genome sequencing

The Generay Genomic DNA Miniprep Kit (Shanghai Generay Biotech Co., Ltd., Shanghai, China) was used to extract the total genomic DNA of *A. mucicolens* Y3, which was sequenced with both the Illumina HiSeq 2500 and PacBio RS II platforms by Shanghai Sunny Biotechnology Co., Ltd. (Shanghai, China), and the read lengths were pE150 and 10 kb with coverage of 150× each, respectively. The PacBio long reads were assembled by Unicycler v0.4.8 (Wick et al., 2017), and the quality of the draft genome assembly was enhanced by Pilon by mapping Illumina short reads to the assembly to correct any misassembled bases (Walker et al., 2014). Genes were predicted and annotated by using Prokka v1.14.6 (Seemann, 2014). DIAMOND v2.0.11 (Buchfink et al., 2021) with an e-value cutoff of 1e-5 was utilized to search the predicted proteins in the NCBI nonredundant protein database. The Comprehensive Antibiotic Resistance Database (CARD)^1^ and Resistance Gene Identifier v5.2.02 were used to annotate the resistance genes (McArthur et al., 2013). Using the NCBI 16S ribosomal RNA database^2^, 16S rRNA gene homology analysis was carried out. The NCBI database^3^ and the gcType database^4^ (Shi et al., 2021) were used to determine the ANI using FastANI (Jain et al., 2018). Multiple sequence alignment of AMZ-1 and other β-lactamases was carried out using MAFFT v7.490 (Katoh and Standley, 2013), and the results were visualized using the msa package in R (Bodenhofer et al., 2015). A neighbor-joining phylogenetic tree including AMZ-1 and other functionally characterized β-lactamases from the Beta-Lactamase DataBase^5^ and UniProtKB/Swiss-Prot Database^6^ was reconstructed using MEGA11 (Kumar et al., 2018). The phylogenetic tree was visualized using the online tool iTol (Letunic and Bork, 2007). The figures of the genetic environments surrounding the *bla*_AMZ–1_ and *bla*_AMZ–1_(-like) genes were generated by clinker v0.0.24 (Gilchrist and Chooi, 2021). The molecular weight and pI value were predicted using ProtParam. GNU Parallel (Tange, 2021).

### Cloning of the *bla*_AMZ–1_ gene and the expression and purification of AMZ-1

Using *A. mucicolens* Y3 genomic DNA as a template for polymerase chain reaction (PCR), the *bla*_AMZ–1_ gene was cloned. The nucleotide sequence of *bla*_AMZ–1_ with its upstream promoter region was amplified by 2× Phanta Max Master Mix (Vazyme, Nanjing, China) (Table 3). The cloning vector pUCP24 was digested with both *Bam*HI and *Xba*I (Takara Bio Inc., Dalian, China) and purified by the FastPure Gel DNA Extraction Mini Kit (Vazyme, Nanjing, China), and the PCR product was also treated with the same procedures as the vector pUCP24. The ligation of the target gene into the linear vector was performed using a T4 DNA ligase cloning kit (Takara Bio Inc., Dalian, China). The recombinant plasmid (pUCP24-pro-*bla*_AMZ–1_) was transformed into *E. coli* DH5α by the calcium chloride method. Bacterial colonies were cultured on LB agar plates supplemented with gentamicin (40 μg/mL). The recombinant plasmid was verified by both PCR and PCR product Sanger sequencing (Shanghai Sunny Biotechnology Co., Ltd., Shanghai, China).

**TABLE 3:** Primers used for cloning the *bla*_AMZ–1_ gene.

Primer^a^	Sequence (5′–3′)	Restriction endonuclease	Vector	Annealing temperature (°C)	Amplicon size (bp)
pro- *bla*_AMZ–1_-F	CGCGGATCCGGAAGGGTCGACGGCGTGGATCGATGCA	*Bam*HI	pUCP24	67	1,447
pro- *bla*_AMZ–1_-R	CTAGTCTAGAGAATTCGATGCGCGGTCCATGGCCAG	*Xba*I			
orf- *bla*_AMZ–1_-F	CGCGGATCCGACGACGACGACAAGTCCGAGCTGCCCTACA TCGACAGCGTCGTGA	*Bam*HI + DDDDK	pColdI	67	1,116
orf- *bla*_AMZ–1_-R	CCCAAGCTTCGGCAGCAACGACGACAGCGCCATCAACG	*Hind*III			

^a^Primers starting with “pro” were used to clone the *bla*_AMZ–1_ gene with its upstream promoter region; primers starting with “orf” were used to clone the ORF of the *bla*_AMZ–1_ gene.

Similar to the cloning procedures, the ORF of *bla*_AMZ–1_ without a signal peptide sequence was cloned (Table 3). Using pColdI as an expression vector, the recombinant plasmid (pColdI-orf-*bla*_AMZ–1_) was transformed into *E. coli* BL21 (DE3). The transformants were cultured on LB agar plates supplemented with ampicillin (100 μg/mL). For the expression of AMZ-1, the recombinant [pColdI-*bla*_AMZ–1_/BL21 (DE3)] was cultured overnight in LB medium, diluted 100-fold in fresh medium with ampicillin (100 μg/mL) and incubated at 37°C and 220 rpm for approximately 2–3 h, until the OD_600_ reached 0.6. After incubation in an ice bath for > 30 min, isopropyl-β-d-thiogalactopyranoside (IPTG, Sigma Chemicals Co., St. Louis, MO, USA) was added at a final concentration of 1 mM, and then another incubation was performed at 16°C for 16 h. The bacteria were collected by centrifugation (8,000 rpm, 4°C, 10 min) using an ultrasonic bacterial crusher, followed by centrifugation (12,000 rpm, 4°C, 30 min) for supernatant collection. The AMZ-1 protein with a his-tag was purified by nickel affinity chromatography with BeyoGold his-tag purification resin (Beyotime, Shanghai, China) and then digested with Enterokinase (GenScript, Nanjing, China) for 36 h at 37°C to remove the his-tag. The protein was identified by SDS-PAGE using a 12% acrylamide separation gel and Coomassie blue G-250 staining. The protein concentration was determined by a BCA kit (Beyotime, Shanghai, China) at 562 nm.

### Enzyme kinetic analysis

Kinetic analysis was performed as previously reported (Bush et al., 1993). Purified AMZ-1 β-lactamase and β-lactam antibiotics were mixed and examined using a UV–VIS spectrophotometer (SpectraMax M5, Molecular Devices, USA) at 37°C in 10 mM phosphate buffer (pH 7.0) in a final reaction volume of 200 μL. The detection wavelength was derived from the maximum absorption wavelength reported in the literature. The steady-state kinetic parameters (*k*_*cat*_ and K_*m*_) were determined by nonlinear regression of the initial reaction rates with the Michaelis-Menten equation in Prism (version 8) software (GraphPad Software, San Jose, CA, USA).

The β-lactamase inhibitors avibactam and tazobactam were used at various concentrations, and nitrocefin (final concentration at 100 μM) was added as a substrate. The samples were preincubated with the purified AMZ-1 β-lactamase for 5 min at 37°C before the addition of substrate, and immediately after the addition of nitrocefin, they were scanned at the optimum wavelength for nitrocefin for 2 min. The inhibitor concentration required to reduce the hydrolysis of 100 μM nitrocefin by 50% (IC50) was determined by nonlinear regression with the log (inhibitor) vs. response (three parameters) in Prism (version 8) software (GraphPad Software, San Jose, CA, USA).

### Accession numbers

The complete chromosome and *bla*_AMZ–1_ gene sequences of *A. mucicolens* Y3 have been submitted to GenBank, and the accession numbers are CP123364 and OQ948417, respectively.

## Results and discussion

### Genome characteristics and resistance profile of *A. mucicolens* Y3

The isolate *A. mucicolens* Y3 was obtained from an anal swab of a goose on a farm in Wenzhou, Zhejiang Province, China. The 16S RNA gene homology analysis showed that the 16S RNA gene of *A. mucicolens* Y3 shared the closest identity (100% coverage and 99.87% identity) with that of *A. mucicolens* LMG 26685 (GCA_902859725.1). Moreover, the ANI analysis revealed that isolate Y3 shared the highest ANI (96.96%) with *A. mucicolens* LMG 26685. ANI is the gold standard for determining species classification, and the cutoff score (≥ 95%) is the threshold to classify the species of a certain bacterium using a comparison between the unclassified bacterium genome and all the bacterial genomes available in public databases (Richter and Rosselló-Móra, 2009). Ultimately, isolate Y3 was assigned to the species *A. mucicolens* and designated *A. mucicolens* Y3.

The *A. mucicolens* Y3 genome consists of a circular chromosome (plasmid-free). The chromosome is approximately 5.68 Mb in length, encoding 5,219 ORFs with an average GC content of 65.90% (Table 4). A search of the NCBI nucleotide database showed that there are 400 genome sequences of the genus *Achromobacter*, of which 14 belong to species *A. mucicolens* (in addition to *A. mucicolens* Y3 in this study). Among the 14 *A. mucicolens* genome sequences, five are from humans, eight are from the environment (including four from sinks, three from soils and one from sediment), and only one is from an animal (cattle). The genome sizes range from 5.6 to 6.4 Mb, with most between 5.9 and 6.3 Mb, and most of the genomes are larger than that of *A. mucicolens* Y3 (Supplementary Table 1).

**TABLE 4:** General features of the *A. mucicolens* Y3 genome.

	Chromosome
Size (bp)	5,682,553
GC content (%)	65.90
CDS	5,219
Known proteins	4,244 (81.32%)
Hypothetical proteins	975 (18.68%)
Protein coding (%)	97.97
Average ORF length (bp)	982.9
Average protein length (aa)	328.5
tRNAs	55
rRNA operators	(16s–23s–5s–5s) ×1, (16s–23s–5s) ×3

In this study, antibiotic susceptibility testing showed that *A. mucicolens* Y3 exhibited high MIC levels for several β-lactam antibiotics, such as cephalothin (> 1,024 μg/mL), penicillin G (1,024 μg/mL), cefotaxime (> 256 μg/mL), cefazolin (128 μg/mL), amoxicillin (64 μg/mL), and aztreonam (64 μg/mL) (Table 2). *A. mucicolens* Y3 was susceptible to piperacillin (< 0.25 μg/mL), which is consistent with the susceptibility of other *Achromobacter* strains (Liu et al., 2016). *Achromobacter* strains have exhibited resistance to narrow-spectrum penicillins, first- and second-generation cephalosporins, ceftriaxone, cefotaxime, and aztreonam, whereas they may still be susceptible to ceftazidime, cefepime, piperacillin, and carbapenem (Isler et al., 2020). Multidrug efflux pumps and chromosomal OXA-114-like β-lactamases are recognized as the primary resistance mechanisms of *Achromobacter* species (Isler et al., 2020). Recently, with whole-genome sequencing, the molecular resistance mechanism of an *A. mucicolens* isolate was determined, wherein 208 drug resistance-related genes, including 25 putative β-lactamase genes, were predicted in the genome (Al-Asadi et al., 2022).

In the annotation results of the whole genome of *A. mucicolens* Y3, none of the predicted ORFs with ≥ 80% amino acid identity with the functionally characterized β-lactamase genes were identified. Instead, three predicted β-lactamase genes with identities between 50.0 and 30.0% with functionally characterized β-lactamase genes were present. They included a *bla*_SRT–C2_-like gene, a *bla*_OXA–656_-like gene, and a *bla*_NmcR_-like gene that shared amino acid identities of 50.0, 39.8, and 32.4% with SRT-C2 (AAQ90024), OXA-656 (WP_122630843), and NmcR (CAA79966), respectively. To determine whether any of them was a functional β-lactam antibiotic resistance gene, the gene with the highest identity (*bla*_SRT–C2_-like) was cloned first, and its resistance function was determined. It was interesting to find that this *bla*_SRT–C2_-like gene (designated *bla*_AMZ–1_ in this work) was indeed functional.

### *Bla*_AMZ–1_ confers resistance to penicillins and cephalosporins

For analysis of the potential function of *bla*_AMZ–1_, the *bla*_AMZ–1_ gene with its promoter region was cloned, and a recombinant plasmid (pUCP24-*bla*_AMZ–1_) was transformed into *E. coli* DH5α. The transformant (pUCP24-*bla*_AMZ–1_/DH5α) exhibited resistance to some penicillins and first- and second-generation cephalosporins. Compared with the control strain (pUCP24/DH5α), the transformant exhibited an increase in the MICs of a variety of β-lactam antimicrobials, including amoxicillin, penicillin G, ampicillin, cephalothin and cefoxitin, the MICs of which increased 64-, 64-, 8-, > 4- and 4-fold, respectively. In addition, the MICs of some antimicrobials, such as aztreonam, cefazolin, and ceftriaxone, increased two-fold each for the transformant compared the control strain. When used in combination with the β-lactamase inhibitors tazobactam and avibactam, both piperacillin (piperacillin + tazobactam) and aztreonam (aztreonam + avibactam) exhibited a 4- and > 8-fold decrease in MIC. The results suggested that the novel β-lactamase resistance gene *bla*_AMZ–1_ may play a role in the β-lactam antibiotic resistance of *A. mucicolens* Y3.

As mentioned above, among all the functionally characterized β-lactamases, *bla*_AMZ–1_ showed 49.0% similarity (50.0% identity and 98.0% coverage) with *bla*_SRT–C2_. Similar to *bla*_AMZ–1_ and compared to the control, *bla*_SRT–C2_ increased MICs by more than four-fold for amoxicillin, cefoxitin and cephalothin (Mammeri et al., 2004). However, *bla*_SRT–C2_ had a wider antibiotic resistance spectrum for second- and third-generation cephalosporins, and it also conferred resistance to cefotaxime, ceftazidime and ceftriaxone and showed much higher MICs for some β-lactam antimicrobials than *bla*_AMZ–1_, such as cefotaxime (16 vs. 0.06 μg/mL) or ceftazidime (512 vs. < 0.015 μg/mL). *Bla*_SRT–C1_ showed much higher MICs for cefazolin (512 vs. 2 μg/mL) and piperacillin (32 vs. 1 μg/mL) than *bla*_AMZ–1_ (Raimondi et al., 2001). *Bla*_LR13–1_ showed the same MICs increase for ampicillin (eight-fold) and cefoxitin (four-fold) as *bla*_AMZ–1_ and a lower MIC for amoxicillin than *bla*_AMZ–1_ (8 vs. 64 μg/mL) (Matsumura et al., 1998). The reason for the difference in MICs may the structural variations between the gene sequences.

To further validate the enzyme’s function, the kinetic parameters of AMZ-1 β-lactamase were analyzed (Table 5). The purified AMZ-1 from the recombinant [pColdI-*bla*_AMZ–1_/BL21(DE3)] exhibited hydrolytic activities against some penicillins (penicillin G and amoxicillin) and some cephalosporins (cephalothin and cefoxitin). Cephalothin showed the highest *K*_m_ value among all the tested substrates, while amoxicillin showed the lowest. However, the results of the enzyme kinetic hydrolytic activity test were not completely consistent with the MIC changes of the recombinant strain (pUCP24-*bla*_AMZ–1_/DH5α) in the antimicrobial susceptibility test (Table 2). Although the recombinant strain (pUCP24-*bla*_AMZ–1_/DH5α) exhibited an eight-fold increase in the MIC of ampicillin, the hydrolytic activity of AMZ-1 for this antimicrobial was not detectable. This result may be attributed to differences in activity between *in vitro* and *in vivo* conditions. β-Lactamase may be overproduced *in vivo* under antibiotic pressure and in *in vitro* experiments; despite several attempts, sufficient amounts of β-lactamase could not be enriched to observe the corresponding antibiotic hydrolysis (Pérez-Llarena et al., 2014). A similar situation has been reported in a previous study for CTX-M-37. Although the recombinant strain exhibited a four-fold increase in the MIC of ceftazidime, the hydrolytic activity of the β-lactamase could not be reliably determined with enzyme concentrations up to 253 nM in the reaction mixture (El Salabi et al., 2012). The turnover (*k*_cat_) values of penicillin G (3.93 s^–1^) and cephalothin (5.88 s^–1^) were approximately 50-fold higher than those of amoxicillin (9.47 × 10^–2^ s^–1^) and cefotaxime (8.05 × 10^–2^ s^–1^). Compared with SRT-C2, AMZ-1 showed stronger hydrolytic activities (*k*_cat_/*K*_m_) for cefoxitin (4.54 vs. 2 mM^–1^⋅s^–1^), while SRT-C2 showed higher hydrolysis activity than AMZ-1 for other antibiotics, such as cephalothin (48,000 vs. 10.4 mM^–1^⋅s^–1^), amoxicillin (10 vs. 6.78 mM^–1^⋅s^–1^) and penicillin G (5,000 vs. 36.3 mM^–1^⋅s^–1^) (Mammeri et al., 2004). In addition, SRT-C2 and AMZ-1 had the same *k*_cat_ values for amoxicillin (0.01 s^–1^) and cefoxitin (0.01 s^–1^). The IC50 (50% inhibitory concentration) of β-lactamase inhibitors showed that the AmpC β-lactamase inhibitor avibactam had a conspicuous inhibitory effect on AMZ-1 (IC50: 819.67 × 10^–6^ μM); however, the class A β-lactamase inhibitor tazobactam showed a much weaker inhibitory effect on AMZ-1 (IC50: 1,094.97 μM) (Bush and Bradford, 2016).

**TABLE 5:** Kinetic parameters of the purified AMZ-1 β-lactamase for various β-lactam antibiotics.

Substrate	*K*_m_ (μM)	*k*_cat_ (s^–1^)	*k*_cat/_K_m_ (mM^–1^⋅s^–1^)
Cephalothin	564.23 ± 56.35	5.88 ± 0.39	10.4 ± 0.04
Amoxicillin	14.65 ± 1.274	(9.47 ± 0.50) × 10^–2^	6.78 ± 1.65
Penicillin G	108.84 ± 8.12	3.93 ± 0.13	36.3 ± 0.15
Cefoxitin	18.16 ± 3.88	(8.05 ± 1.37) × 10^–2^	4.54 ± 0.66
Ampicillin	NH*	NH*	NH*

*NH, no detectable hydrolysis.

### Molecular characteristics and phylogenetic relationship of AMZ-1 to its relatives

The novel β-lactamase gene *bla*_AMZ–1_ is 1,191 bp in size and encodes a premature protein of 396 amino acids. The protein has a predicted molecular weight of 42.82 kDa and a predicted pI of 9.42. There is a signal peptide cleavage site between glycine and arginine at amino acid residues 26 and 27. The molecular weight of the mature protein without the signal peptide is 40.06 kDa.

**FIGURE 1:**
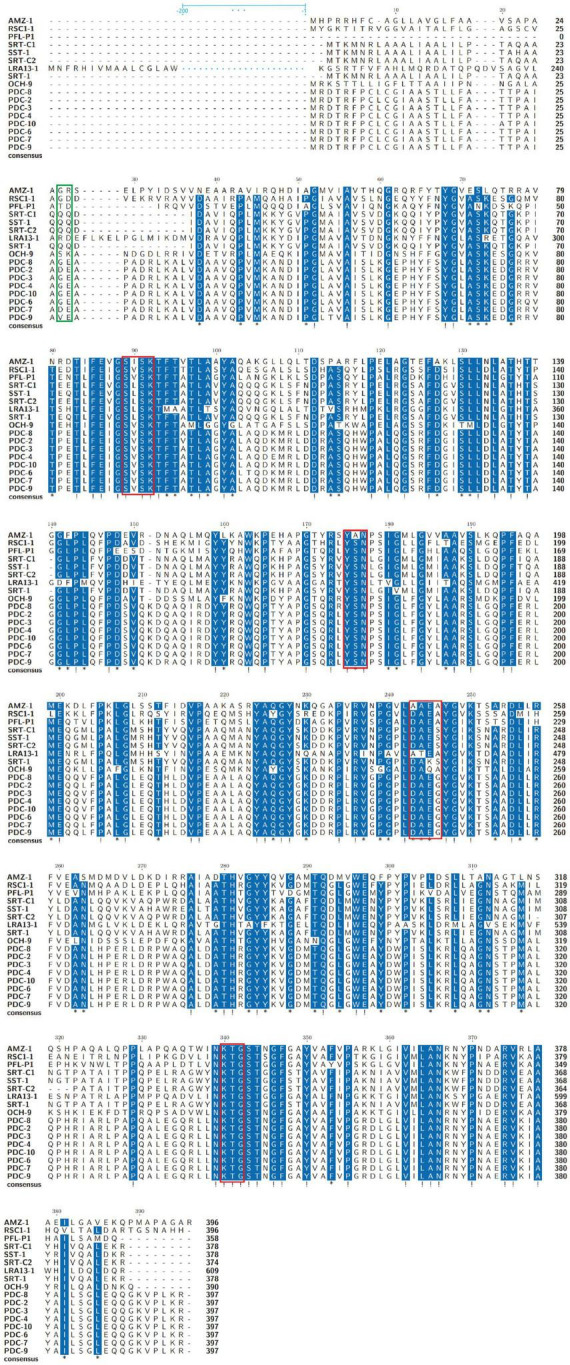
Multiple amino acid sequence alignment of AMZ-1 and other class C β-lactamases. Exclamations indicate fully conserved residues; asterisks indicate strongly similar residues; gaps are represented using hyphens. The green frame indicates the peptide cleavage site, and the red frames indicate four conserved motifs. Two hundred amino acids are omitted and are indicated by blue ellipses for LRA13-1.

To analyze the structural characteristics of the novel β-lactamase AMZ-1, 16 functionally characterized homologs (identity > 47.0%) were retrieved from the β-lactamase database. Alignment of the deduced amino acid sequence of AMZ-1 against the 16 β-lactamases revealed that four structural elements that are essential for the activity of the class C β-lactamases are conserved in AMZ-1 and the other β-lactamases. These include the characteristic catalytic residues of the serine active site of β-lactamases, S-I-S-K (serine-isoleucine-serine-lysine) at positions 89–92 and the typical class C β-lactamase motifs Y-A-N (tyrosine-alanine-asparagine) at positions 175–177, A-A-E-A (alanine-alanine-glutamic-alanine) at positions 243–246, and K-T-G (lysine-threonine-glycine) at positions 340–342 (Figure 1).

**FIGURE 2:**
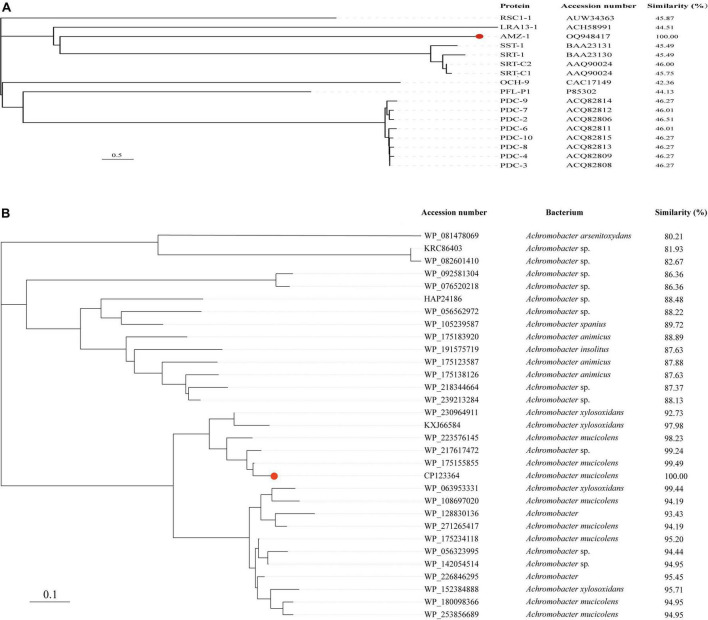
A phylogenetic tree showing the relationship of AMZ-1 to other functionally characterized AmpC β-lactamases **(A)** and to other putative β-lactamases (> 80% similarity) **(B)**. AMZ-1 is highlighted in red.

Further phylogenetic analysis of AMZ-1 against the 16 functionally characterized β-lactamases revealed that AMZ-1 appeared as a new branch and was located relatively close to the SST-1 and SRT proteins (including SRT-1, SRT-C1 and SRT-C2) (Figure 2A). This result suggested that AMZ-1 constitutes a novel lineage of the β-lactamase family. Analysis of the distribution of the *bla*_AMZ–1_ homologous genes revealed that 31 functionally uncharacterized proteins with > 80% global amino acid sequence similarity with AMZ-1 were present in the NCBI database, and they were all from the genus *Achromobacter*. Apart from 13 sequences without specific species classification, most sequences were from *A. mucicolens* (7 sequences). The closest relative to AMZ-1 was a putative class C β-lactamase (WP_175155855) from *A. mucicolens* sharing an amino acid sequence similarity of 99.49% (99.49% identity and 100% coverage). The phylogenetic analysis also confirmed that this protein shared the closest evolutionary relationship with AMZ-1 (Figure 2B).

### Genetic environments of the *bla*_AMZ–1_ and *bla*_AMZ–1_-like genes

**FIGURE 3:**
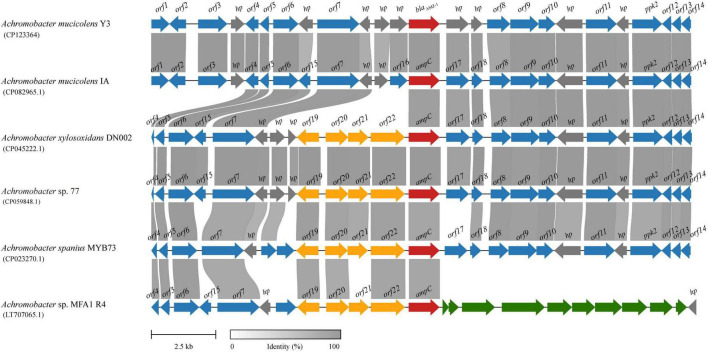
Genetic environment of the *bla*_AMZ–1_ and *bla*_AMZ–1_-like genes. The regions with ≥ 80% amino acid identity are colored gray. AmpC β-lactamases are colored red and the other genes are colored based on gene function. *hp*, hypothetical protein; *ppk2*, polyphosphate kinase 2; other *orfs* without direct gene names are listed in Supplementary Table 2.

To analyze the genetic environments of *bla*_AMZ–1_ and its relatives, the sequences containing a *bla*_AMZ–1_-like gene at the center that shared ≥ 85.0% amino acid identity and ≥ 95% coverage with *bla*_AMZ–1_ were retrieved from the NCBI nucleotide database. Finally, a total of five sequences approximately 20 kb in length with *bla*_AMZ–1_-like genes at the centers were retained for further analysis. All five sequences were from the genus *Achromobacter*, including one (*A. mucicolens* IA) from the same species as the one characterized in this work, *A. mucicolens*. The other four included *A. xylosoxidans* (*A. xylosoxidans* DN002), *A. spanius* (*A. spanius* MYB73), and 2 strains with unclassified species (*Achromobacter* sp. 77 and *Achromobacter* sp. MFA1 R4) (Figure 3). No mobile genetic elements were predicted in the regions surrounding these sequences. Comparative genomic analysis of the 6 sequences (including the sequence characterized in this work) revealed that the structure of one sequence from the same species (*A. mucicolens* IA) was most similar to the structure of the sequence in this work, with 100% coverage and 97.39% identity. Among the other four sequences, three shared similar downstream regions with the sequence characterized in this work; however, the downstream region of the last sequence was completely different from the others. In the upstream regions, the four sequences showed similar structures but were different from the other two (the sequence characterized in this work and *A. mucicolens* IA), and they had 4 to 5 extra ORFs inserted adjacent to the *bla*_AMZ–1_-like genes.

## Conclusion

In this work, a novel β-lactamase gene designated *bla*_AMZ–1_ encoded in the chromosome of *A. mucicolens* Y3 isolated from a goose was characterized. AMZ-1 shared the highest amino acid identity of 50.0% with the functionally characterized AmpC enzyme SRT-C2. The cloned *bla*_AMZ–1_ gene showed resistance against some β-lactam antibiotics, including amoxicillin and cephalothin, and the β-lactamase AMZ-1 exhibited a similar substrate spectrum response. Similar to other AmpC β-lactamases, AMZ-1 is strongly inhibited by avibactam but much less strongly inhibited by tazobactam. Closer homologs of *bla*_AMZ–1_ tend to appear in the different species of the genus *Achromobacter*. The identification and characterization of the novel resistance gene will help in the selection of effective antimicrobials to treat infections caused by opportunistic pathogens that carry the novel resistance gene.

## Data availability statement

The datasets presented in this study can be found in online repositories. The names of the repository/repositories and accession number(s) can be found in this article/Supplementary material.

## Ethics statement

This study used strains obtained from an anal swab of a goose on an animal farm in Wenzhou. The owner of the farm was informed of the study and expressed approval for the sampling of animals. All experimental procedures involving animals were approved by the Animal Welfare and Ethics Committee of Wenzhou Medical University, Zhejiang Province, China.

## Author contributions

HZ, KL, QB, and DL: conceived and designed the experiments. YZ, JZ, GZ, NL, YS, JL, and TZ: performed the experiments. YZ, XZ, QL, and XL: data analysis and interpretation. YZ, QB, and DL: drafting of the manuscript. All authors contributed to the article and approved the submitted version.

## Footnotes

1
http://arpcard.mcmaster.ca

2
https://blast.ncbi.nlm.nih.gov

3
https://www.ncbi.nlm.nih.gov/

4
https://gctype.wdcm.org/

5
http://www.bldb.eu/

6
https://www.uniprot.org/
