# Supplementary material for: Identification and characterization of a novel β-lactamase gene, blaAMZ–1, from Achromobacter mucicolens
Source: Front Microbiol. 2023 Sep 21;14:1252427. doi: 10.3389/fmicb.2023.1252427 (PMC10552758; doi:10.3389/fmicb.2023.1252427)
Supplement: Supplementary file 1 [file Table_1.docx]

TABLE S1 | Genome sizes and sources of the *A. mucicolens* strains retrieved from the NCBI database.

| **Assembly accession** | **BioSample ID** | **Strain name** | **Isolation source/host** | **Genome size** |
| --- | --- | --- | --- | --- |
| GCF_029837185 | SAMN30525496 | GD04040 | sink | 6.4 Mb |
| GCF_902859995 | SAMEA6797404 | LMG 3415 | human | 6.3Mb |
| GCA_902860005 | [SAMEA6797390](https://www.ncbi.nlm.nih.gov/biosample/SAMEA6797390/) | LMG 26684 | human | 6.3Mb |
| GCF_029838805 | SAMN30525423 | GD03967 | sink | 6.3 Mb |
| GCA_902860105 | SAMEA6797391 | LMG 26686 | human | 6.0 Mb |
| GCA_027941855 | SAMN32638594 | DMF-4 | sediment | 6.0 Mb |
| GCF_024178435 | SAMN28197032 | HR2 | soil | 6.0 Mb |
| GCF_019968625 | SAMN21168694 | IA | human | 5.9Mb |
| GCA_902859725 | SAMEA6647240 | LMG 26685 | human | 5.9Mb |
| CF_003053425 | SAMN08887234 | KCJK8006 | cattle | 5.9Mb |
| GCF_029842925 | SAMN30525211 | GD03755 | sink | 5.8 Mb |
| GCF_029834855 | SAMN30525615 | GD04159 | sink | 5.6 Mb |
| GCF_029256815 | SAMN20393880 | L_E1_T20_A_bin.127 | soil | 5.9Mb |
| GCF_025548485 | SAMN30742219 | CSR5 | soil | 5.9Mb |
