# Supplementary material for: Identification and characterization of a novel β-lactamase gene, blaAMZ–1, from Achromobacter mucicolens
Source: Front Microbiol. 2023 Sep 21;14:1252427. doi: 10.3389/fmicb.2023.1252427 (PMC10552758; doi:10.3389/fmicb.2023.1252427)
Supplement: Supplementary file 2 [file Table_2.docx]

TABLE S2 | Open reading frame gene names in the surrounding environment.

| ***orf*** | **Function** |
| --- | --- |
| *orf*1 | helix-turn-helix transcriptional regulator |
| *orf*2 | hemolysin III family protein |
| *orf*3 | PHB depolymerase family esterase |
| *orf*4 | VOC family protein |
| *orf*5 | DUF1428 family protein |
| *orf*6 | helix-turn-helix domain-containing protein |
| *orf*7 | Methyl-accepting chemotaxis sensor/transducer protein |
| *orf*8 | MetQ/NlpA family lipoprotein |
| *orf*9 | Methionine ABC transporter ATP-binding protein |
| *orf*10 | Methionine ABC transporter permease protein |
| *orf*11 | Methionine gamma-lyase |
| *orf*12 | DOPA 4,5-dioxygenase family protein |
| *orf*13 | Translation initiation factor SUI1-related protein |
| *orf*14 | RNA-binding S4 domain-containing protein |
| *orf*15 | GNAT family N-acetyltransferase |
| *orf*16 | SMI1/KNR4 family protein |
| *orf*17 | DUF2242 domain-containing protein |
| *orf*18 | CopD family protein |
| *orf*19 | AraC family transcriptional regulator |
| *orf*20 | fumarylacetoacetate hydrolase family protein |
| *orf*21 | alpha/beta fold hydrolase |
| *orf*22 | amidohydrolase family protein |
